# Localized versus 360-degree laser photocoagulation with limited pars plana vitrectomy in the management of primary rhegmatogenous retinal detachment

**DOI:** 10.1186/s12886-022-02614-5

**Published:** 2022-10-07

**Authors:** Na-Kyung Ryoo, So Yeon Kim, Se Joon Woo, Kyu Hyung Park

**Affiliations:** 1 Department of Ophthalmology, Seoul National University College of Medicine, Seoul, Korea; 2 Department of Ophthalmology, Veterans Health Service Medical Center, Seoul, Korea; 3 Department of Ophthalmology, Seoul National University Bundang Hospital, 82 gumiro 173, Bundang-gu, Seongnam, Korea; 4 Department of Ophthalmology, Seoul National University Hospital, 101 Daehak-ro, Jongno-gu, 03080 Seoul, Korea

**Keywords:** Rhegmatogenous retinal detachment, Endolaser, Limited vitrectomy, Pars plana vitrectomy, Laser photocoagulation, Focal laser photocoagulation, 360-degree laser photocoagulation

## Abstract

**Background:**

To compare the efficacy of intraoperative localized and 360-degree laser photocoagulation in 23-gauge limited pars plana vitrectomy (PPV) for rhegmatogenous retinal detachment (RRD).

**Methods:**

This retrospective, comparative, consecutive, interventional study included 155 eyes of 155 patients who underwent primary repair of RRD utilizing 23-gauge PPV with at least six months of follow up. Medical records were retrospectively reviewed, and the corresponding demographic data, preoperative ophthalmic features, surgical management, and postoperative course were recorded. Main outcome measures included single surgery anatomical success, pre- and post-operative visual acuity, and complications.

**Results:**

Eighty-three patients (group A) received localized laser photocoagulation in PPV, while the remaining 72 patients (group B) received underwent circumferential 360-degree laser photocoagulation in PPV. Two skilled-surgeons performed all the surgeries, and 23-gauge PPV instrumentation, a wide-angle viewing system, endolaser photocoagulation, and gas tamponade were used in each case. No significant difference was identified in baseline characteristics. The single surgery anatomical success rate was 96.4 % in group A, and 95.8 % in group B, showing no significant difference (*p = 1.00*). Primary anatomical failure was caused by re-detachment due to break in 2 eyes in each group (no new break 1 eye, new break 1eye in group A, 2 eyes with no new break in group B), and proliferative vitreoretinopathy in 1 eye in each group. Other complications were epiretinal membrane in 7 eyes (3 in group A, 4 in group B), and macular hole in 1 eye in group B. There were no differences in pre- and post-operative best-corrected visual acuity (BCVA) as well as BCVA improvement (*p*=0.144, *p*=0.866 and *p*=0.263, respectively).

**Conclusion:**

Localized laser photocoagulation showed no difference in anatomic and visual outcome in RRD patients, when compared with 360-degree laser photocoagulation in limited PPV. Routine circumferential 360-degree laser photocoagulation may not be necessary in vitrectomy surgery for primary rhegmatogenous retinal detachment without severe PVR.

## Background

Over the past decades, the surgical outcomes in the repair of rhegmatogenous retinal detachment (RRD) have improved immensely [1, 2]. The development and sophistication of pars plana vitrectomy (PPV) instruments, wide-angle viewing systems, laser photocoagulation (endolaser), and tamponade materials have enhanced our ability to successfully treat RRD [3].

Among these procedures, laser photocoagulation is considered essential in that it can rapidly enhance chorioretinal adhesion [4]. However, there is controversy regarding the optimal usage of laser photocoagulation for primary repair of rhegmatogenous retinal detachment. Localized laser photocoagulation may be applied to treat the retinal break or weak retinal area, such as lattice degeneration. On the other hand, 360-degree laser photocoagulation may be preferred as a prophylactic measure, in addition to treating the known lesion, to prevent any recurrence of retinal detachment. Some studies report better visual and anatomical outcome with 360° laser photocoagulation [5–8], while others find less benefit in prophylactic procedures [9, 10].

As controversies exists with the majority of the vitreoretinal surgeons performing laser photocoagulation at their own discretion, based on personal experience and preference, the purpose of this study was to compare the efficacy of intraoperative localized and 360-degree laser photocoagulation in sutureless 23-gauge pars plana vitrectomy (PPV) for rhegmatogenous retinal detachment (RRD).

## Methods

This retrospective, comparative, consecutive, interventional study was conducted in accordance with the Declaration of Helsinki. This study was approved by the Institutional Review Board of Seoul National University Bundang Hospital (IRB No. B1810-501-101). Due to the retrospective design of the study and the use of deidentified patient information, the Institutional Review Board of Seoul National University Bundang Hospital also waived the need for written informed consent.

### Subjects

The medical records of all consecutive patients admitted for surgery for primary rhegmatogenous retinal detachment (RRD) between January 2012 and December 2015 at the Department of Ophthalmology of Seoul National University Bundang Hospital, Seongnam, Korea, were reviewed. All patients underwent sutureless 23-gauge PPV surgery, including photocoagulation (either localized or 360-degree) and intravitreal gas tamponade (18% SF6 or 14% C3F8 gas) as their initial treatment.

Included in this study were patients, who underwent PPV for the treatment of RRD with a minimum follow-up period of 6 months. Exclusion criteria were 1) history of ocular surgery (except cataract surgery), 2) proliferative vitreoretinopathy (PVR) worse than grade C (Retina Society Terminology Committee Classification) [11], 3) concomitant scleral buckling, 4) non-primary RRD such as recurrent RD and non-rhegmatogenous RD (e.g. serous RD, tractional RD), 5) Other severe vision-impairing diseases (e.g., advanced glaucoma, optic neuropathy etc.)

### Collection of data

The following information was obtained from the patient’s electronic medical record: age, gender, comorbidity (diabetes and hypertension), laterality of eye in which surgery was performed, axial length, lens status, pre- and postoperative best-corrected visual acuity (BCVA), and follow-up period. Detailed information on the status of retinal detachment were also obtained, such as extent of RD, macula status (on/off), number and location of retinal breaks, including lattice degeneration. Type of re-operation, as well as post-operative complications were collected.

### Surgical technique

Surgical procedures were performed by two skilled vitreoretinal surgeons (S.J.W and K.H.P), under either general anesthesia or subtenon anesthesia. Phacoemulsification was carried out when there were marked lens opacities that potentially compromised the patient’s visual acuity. A transconjunctival sutureless PPV was performed using a three-port 23-gauge vitrectomy system (Alcon Accurus or Constellation instruments (Alcon Laboratories, Fort Worth, TX)). A wide-angle viewing system—BIOM non-contact panoramic viewing system (Oculus, Wetzlar, Germany) or RESIGHT 700 Fundus viewing system (Carl Zeiss Meditec AG, Jena, Germany)—was used during vitrectomy. Core vitrectomy was performed followed by removal of peripheral vitreous traction around the retinal tears, with or without heavy liquid (perfluorodecalin, DK-Line®, Bausch&Lomb Inc. New York, USA). Peripheral vitreous was sufficiently removed to create enough space for gas tamponade under wide-angle viewing system. Vitreous shaving was done in areas anterior and adjacent to the tear. However, peripheral vitreous shaving with scleral depression was not performed in the periphery irrelevant to the tear. All the tears were marked with endodiathermy and internal subretinal fluid drainage was performed through the existing retinal breaks without any additional retinotomy. Following fluid-air exchange, all retinal breaks and lattice degeneration were barricaded with endolaser photocoagulation. 360° prophylactic laser photocoagulation was performed at the surgeons’ discretion and preference. Eyes with tears in all four quadrants or with diffuse lattice degeneration were more subject to receive 360-degree laser photocoagulation. After completion of laser photocoagulation, either 18% sulfurhexafluoride (SF_6_) or 14% perfluoropropane (C_3_F_8_) was used for gas tamponade. All patients were asked to avoid supine position and maintain face-down position for 7 days after surgery.

### Primary outcome measures

The main outcomes measured were the single surgery anatomic reattachment rate and visual outcome. Anatomical success of surgery was defined as complete disappearance of subretinal fluid and flattening of retina posterior to the demarcation laser photocoagulation. Clinically significant complications that required additional intervention, such as PVR, epiretinal membrane, and macular hole were also recorded.

### Statistical analysis

Statistical analyses were performed using SPSS version 24.0 (SPSS, Inc., Chicago, IL). Data are reported as the mean ± standard deviation or n (%). Demographic and clinical data of two groups were compared with Student t-test or Chi-square test whenever appropriate. For statistical analysis Snellen BCVA values were converted to logMAR units. *P*-value of <0.05 was considered statistically significant.

## Results

**Fig. 1:**
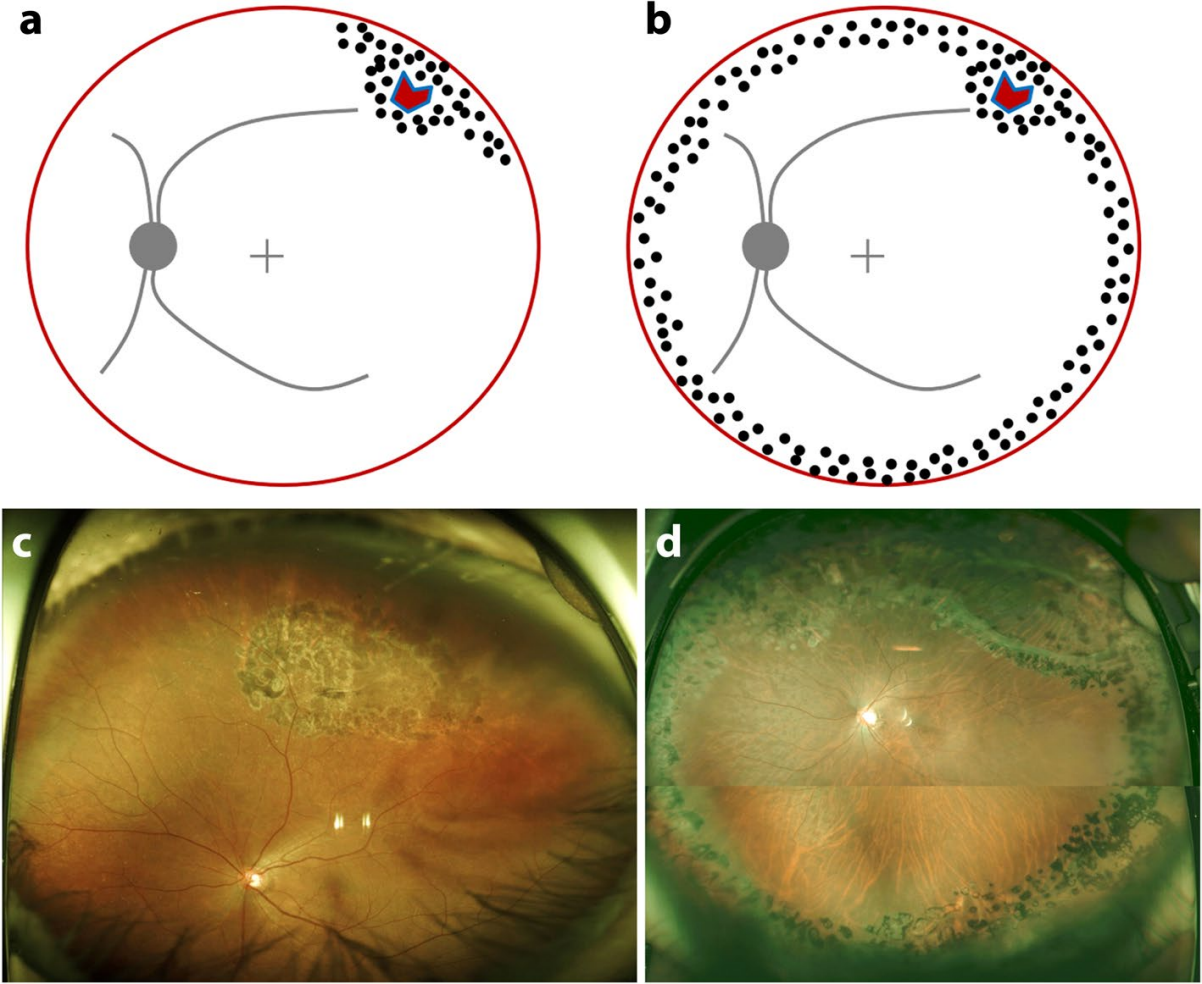
Schematic and representative cases of laser photocoagulation after pars plana vitrectomy for rhegmatogenous retinal detachment are shown in illustration and postoperative wide-field fundus photograph. Localized laser photocoagulation (group A) (**a**, **c**), 360-degree laser photocoagulation (group B) (**b**, **d**)

A total of 155 eyes of 155 patients were included in this study: 83 eyes in group A (localized laser group) and 72 eyes in group B (360-degree laser group) (Fig. 1). The mean age of the patients was 55.8 ± 11.2 years (56.5 ± 8.9 years in group A and 53.7 ± 12.2 years in group B, *p*=0.12). The mean follow-up period was 21.9 ± 14.7 months. Demographic and baseline clinical characteristics of the two groups of patients are summarized in Table 1. No statistical significance was found between the two groups.

**Table 1:** Demographics & baseline clinical characteristics

	**Group A** **(*n*=83)**	**Group B** **(*n*=72)**	*P-value*
**Gender** (n, (%))			0.13
**Male**	49 (59)	51 (71)	
**Female**	34 (41)	21 (29)	
**Age** (years, mean±SD)	56.5 ± 8.9	53.7 ± 12.2	0.12
**DM** (n, (%))	5 (6)	3 (4)	0.44
**Hypertension** (n, (%))	22 (27)	19 (26)	0.95
**Laterality of eye** (n, (%))			0.15
**Right**	51 (61)	36 (50)	
**Left**	32 (39)	36 (50)	
**Axial length** (mm)	25.2 ± 2.5	25.3 ± 3.7	0.77
**Lens status**			0.43
**Phakic**	60 (72)	56 (78)	
**Pseudophakic**	23 (28)	16 (22)	
**Follow-up period** (mo)	21.9 ± 14.1	21.8 ± 15.5	0.99

Group A, localized laser group; Group B, 360-degree laser group; *DM* diabetes mellitus

There was also no significant difference in the anatomic features of rhegmatogenous retinal detachment (Table 2). Extent of retinal detachment was 5.15 ± 1.7 versus 5.07 ± 2.1 clock-hours in group A and B, respectively (*p*=0.82). Superior breaks were the most common type accounting for 69.9% in group A and 75% in group B (*p*=0.303). The majority of cases had less than three retinal breaks (92.6% in group A, 90.30 % in group B; *p*=0.608). Lattice degeneration, apart from retinal breaks, were accompanied in 48.1% and 52.8% in each group. The average number of breaks located outside the detached retinal area were 0.22 ± 0.7, and 0.3 ± 0.9 in group A and B, respectively (*p*=0.542).

**Table 2:** Preoperative anatomical and intraoperative surgical factors

	**Group A (*n* = 83)**	**Group B (*n* = 72)**	***P-value***
**Extent of RD (clock-hours)**	5.15 ± 1.7	5.07 ± 2.1	0.82
**Macula-status** (n, (%))			0.21
ON	32 (39)	35 (49)	
OFF	51 (61)	37 (51)	
**BREAK area** (n, (%))			0.30
SUP	58 (70)	54 (75)	
INF	16 (19)	10 (14)	
BOTH	7 (8)	6 (8)	
**Retinal breaks within RD area (≤3, over 3,** n, (%)**)**			
1	45 (56)	37 (52)	
2	17 (20)	21 (30)	
3	13 (16)	7 (10)	
> 3	8 (10)	7 (10)	
≤ 3	75 (93)	65 (90)	0.61
**lattice degeneration** (n, (%))	39 (48)	38 (53)	0.57
**Retinal breaks outside RD area** (n)	0.22 ± 0.68	0.30 ± 0.91	0.54
**Tamponade Type** (n, (%))			0.06
SF6	78 (94)	61 (85)	
C3F8	4 (5)	11 (15)	
**Combined cataract surgery** (n, (%))	27 (33)	23 (32)	0.94

Group A, localized laser group; Group B, 360-degree laser group

Approximately, a little over 30 percent of the patients underwent concurrent cataract surgery, with no significant difference among the two groups (Table 2). There was an overall preference of SF6 for gas tamponade (94% in group A and 84.7% in group B), and a preference of C3F8 in group B, although not statistically significant (4.8% vs. 15.3%, *p*=0.06) (Table 2).

All the eyes achieved improved BCVA after surgery (Table 3). No significant differences were found in BCVA improvement (1.01 ± 1.0 logMAR units and 0.82 ± 0.95 logMAR units, respectively, *p*=0.263) and final BCVA (0.14 ± 0.27 logMAR units and 0.15 ± 0.26 logMAR units, respectively, *p*=0.866) between groups A and B.

**Table 3:** Surgical outcomes

	**Group A** **(*n*=83)**	**Group B** **(*n*=72)**	*P-value*
**Visual Outcomes** (logMAR)			
**Preop BCVA**	1.21 ± 0.97	0.97 ± 1.00	0.14
**Final BCVA**	0.14 ± 0.27	0.15 ± 0.26	0.87
**BCVA improvement**	1.01 ± 1.00	0.82 ± 0.95	0.26
**Anatomic Outcomes** (n, (%))			
**Primary reattachment rate**	80 (96)	69 (96)	1.00
**Final reattachment rate**	83 (100)	72 (100)	1.00

Group A, localized laser group; Group B, 360-degree laser group

Single-surgery anatomical success was achieved in 96.4% (80/83) of the patients in group A and 95.8 % (69/72) of the patients in group B (*p*=1.00) (Table 3). Among the three that initially failed for reattachment in group A, were one failure with no additional tears, one with a new tear, and one PVR case (Table 4). In group B, 2 cases had no new breaks but continuous retinal detachment, and one case had PVR. Postoperative complications along the course that required additional surgeries were 3 cases of ERM in group A, 4 cases of ERM and one case of macular hole in group B. Final retinal reattachment was achieved in all eyes (100%) in both groups after an additional partial vitrectomy or encircling (one eye in group A, and two eyes in group B).

**Table 4:** Postoperative complications

		**Group A** **(*n* = 83)**	**Group B** **(*n* = 72)**		*P-value*
Recurred RD	with no new break	1	2		
	new tear	1	0		
PVR		1	1		
ERM		3	4		
MH		0	1		
**total N** (no, (%))		6 (7)	8 (11)		0.4

*RD* retinal detachment, *PVR* proliferative vitreoretinopathy, *ERM* epiretinal membrane, *MH* macular hole

## Discussion

Laser photocoagulation with small-gauge PPV is widely used to treat rhegmatogenous retinal detachment. Prior studies have consistently advocated the benefits of 360- degree laser photocoagulation in terms of higher primary success rate and greater improvement in postoperative BCVA [5–8]. However, with the development and refinement of PPV instruments, not only has the surgical risk dropped in general, but attempts have been made to simplify procedures and diminish unnecessary manipulation. The extent of laser photocoagulation, in such matters, may be revisited.

In this study, we compared the efficacy of PPV with localized photocoagulation versus 360-degree laser photocoagulation in primary RRD. Single-operation success was achieved in 96.4% of the cases which underwent localized photocoagulation, and 95.8% of the cases of PPV with 360-degree laser photocoagulation. This is in accordance with, or higher than previous studies that have reported success rates ranging between 74% and 96% [3, 10, 12, 13]. In a study of 27 RRD eyes, although no control group existed, circumferential 360-degree laser was not performed yet a high surgical success rate was noted [14]. In our comparative study, there was no statistically significant difference in the primary reattachment rate between localized and 360-degree laser photocoagulation, and the final surgical success rate also showed no difference.

Contrary to previous reports [7, 10] noting the difference in BCVA improvement, there was no difference in logMAR BCVA improvement in our study. Considering the postoperative complications, such as ERM, macular hole, etc., 360-degree laser group had a higher fraction of those who underwent additional surgeries (7.2% in localized photocoagulation group versus 11.1% in 360-degree photocoagulation group). This is somewhat consistent with the study done by the primary retinal detachment outcomes (PRO) study group [15], which implicates that 360-degree laser photocoagulation is not associated with better surgical outcomes and may even relate to worse anatomic and visual outcomes.

Vitrectomy, in our study, was confined to what was visualized under the wide-angle viewing system. Scleral indentation for the purpose of complete peripheral vitreous shaving was not performed. In accordance with our study, recent studies have shed light on limited vitrectomy in RRD [14, 16]. Wibbelsman et al. reported a single operation surgical success rate of 88.9% (24 of 27 eyes), and a final reattachment rate of 100% in pseudophakic RRD with no peripheral vitreous shaving [14]. Good anatomical—single surgery success 95%, final reattachment 99% —and visual outcome was also reported by Tabandeh et al. in small-gauge vitrectomy for RRD without any scleral-depressed shaving [16]. Therefore, unlike the common belief that meticulous removal of the peripheral vitreous is essential in reattaching the retina or reducing PVR, total thorough vitrectomy may not be a necessity in all cases.

Routine circumferential (360-degree) laser photocoagulation was applied in Tabandeh et al. which encompassed all severity range of RRD, including cases of advanced PVR and complicated RRD [16]. The authors credit circumferential laser photocoagulation for the relatively high surgical success rate under limited vitrectomy. However, in our study with limited vitrectomy, there were no differences in the surgical outcomes between localized or 360-degree laser photocoagulation group. Thus, in cases of RRD with a low degree of PVR, as in our study, 360-degree laser photocoagulation may not be essential.

A few limitations to consider is the retrospective nature of the design and the possibility of certain selection bias. Since, the decision of the degree of laser to apply was determined by the surgeon’s preference, this may have caused selection bias. 360-degree laser photocoagulation was preferred by one surgeon during a limited period. Cases that were relatively severe or had diffuse lattice degeneration may have had a higher possibility of receiving 360-degree laser photocoagulation. Prospective studies will be necessary for further evaluation on limited vitrectomy with localized laser or 360-degree laser photocoagulation

In conclusion, compared with 360-degree laser photocoagulation, localized laser photocoagulation showed no difference in anatomic and visual outcome in RRD patients. This suggests that localized laser photocoagulation in limited PPV may be as effective as 360-degree laser photocoagulation with limited PPV in primary RRD cases without severe PVR.

## Data Availability

The datasets generated during and analyzed during the current study are not publicly available due to institutional database access restrictions but are available from the corresponding author on reasonable request
